# Formación doctoral en virología para la preparación ante pandemias: una necesidad estratégica para Colombia y Latinoamérica

**DOI:** 10.7705/biomedica.8618

**Published:** 2026-08-20

**Authors:** Diana Marcela Pava-Garzón

**Affiliations:** 1 Directora general, Instituto Nacional de Salud Bogotá, D. C., Colombia Instituto Nacional de Salud Bogotá, D. C. Colombia

La relación entre los virus y la humanidad ha definido, de manera profunda, la historia de las sociedades, la evolución de los sistemas de salud y el desarrollo de la ciencia biomédica. Desde las primeras evidencias de enfermedades eruptivas sugestivas de viruela en las civilizaciones antiguas hasta la pandemia de COVID-19, los virus han demostrado una extraordinaria capacidad de adaptación, permitiéndoles cruzar barreras entre especies, originar epidemias y pandemias, y transformar las dinámicas sociales, económicas y sanitarias a escala global. En este contexto, la permanente emergencia y reemergencia de agentes patógenos virales pone de manifiesto que la formación de talento humano especializado constituye uno de los pilares estratégicos para fortalecer la preparación, la vigilancia y la capacidad de respuesta frente a las amenazas biológicas del presente y del futuro.

La historia de las enfermedades infecciosas ilustra con claridad el impacto que los virus han ejercido sobre la humanidad. Entre ellos, el de la viruela representa uno de los más paradigmáticos de la capacidad devastadora de estos agentes biológicos. Causada por el virus variola (*Variola virus,* VARV), esta enfermedad acompañó a la especie humana durante más de tres milenios, provocando epidemias recurrentes, una elevada mortalidad y profundas consecuencias demográficas, sociales y económicas en todos los continentes. Se estima que solo durante el siglo XX ocasionó cerca de 300 millones de muertes, una cifra superior a la atribuida a numerosos conflictos armados en conjunto. Su erradicación, certificada por la Organización Mundial de la Salud en 1980 tras una de las campañas de vacunación más ambiciosas de la historia, constituye uno de los mayores hitos de la salud pública mundial y el único caso en el que una enfermedad infecciosa humana ha sido eliminada por completo mediante una intervención sanitaria coordinada a nivel global.

Este logro no solo evidenció el poder de la vacunación como herramienta de prevención, sino que también demostró el valor de la cooperación científica internacional, la vigilancia epidemiológica y la formación de profesionales altamente capacitados para enfrentar enfermedades infecciosas. Décadas después, estos mismos principios continúan siendo la base sobre la cual se sustentan las estrategias globales para responder a los desafíos que plantean los virus emergentes y reemergentes, cuya capacidad de evolución exige una permanente preparación científica y técnica.

A pesar de la erradicación de la viruela -uno de los mayores triunfos de la salud pública mundial-, las pandemias virales han continuado representando una amenaza permanente para la humanidad. En los dos últimos siglos, el mundo ha enfrentado sucesivas emergencias sanitarias provocadas por virus respiratorios, entre ellas, la devastadora pandemia de influenza de 1918, que ocasionó decenas de millones de muertes, así como las pandemias de influenza de 1957, 1968 y 2009, y más recientemente, la pandemia de COVID-19. Cada uno de estos episodios puso de manifiesto que la aparición de nuevos virus con capacidad de transmitirse eficientemente entre los seres humanos sigue siendo uno de los mayores desafíos para la salud pública global. Más allá de su impacto clínico, estas pandemias evidenciaron la estrecha interdependencia entre la salud, la economía, la gobernanza y la estabilidad social, reafirmando la necesidad de fortalecer la investigación científica, la vigilancia epidemiológica y la capacidad de respuesta de los sistemas sanitarios.

En este contexto, el comprender la extraordinaria diversidad del mundo viral constituye una prioridad científica. Los virus representan las entidades biológicas más abundantes del planeta y desempeñan un papel esencial en el equilibrio de los ecosistemas y en la evolución de la vida. Se estima que existen alrededor de 10^31^ partículas virales distribuidas en ambientes terrestres, acuáticos y atmosféricos, una cifra que supera ampliamente la de cualquier otra entidad biológica conocida. Sin embargo, el conocimiento sobre esta inmensa diversidad continúa siendo limitado. La clasificación más reciente del *International Committee on Taxonomy of Viruses* (ICTV) reconoce poco más de 17 500 especies virales, una proporción mínima frente al vasto universo viral que permanece inexplorado.

La brecha entre la diversidad estimada y la actualmente caracterizada pone en evidencia uno de los mayores retos de la virología contemporánea. Millones de virus continúan sin ser identificados ni estudiados, muchos de ellos con potencial para afectar la salud humana, animal y ambiental o para desempeñar funciones determinantes en los ecosistemas. Este panorama resalta la necesidad de fortalecer la investigación básica y aplicada, de ampliar las capacidades de vigilancia genómica y de consolidar programas especializados de formación que permitan anticipar y comprender futuras amenazas biológicas desde el enfoque integral de *Una salud* (*One health*).

La enorme diversidad viral también se expresa en una complejidad estructural y genética excepcional. Los virus pueden poseer genomas de ADN o ARN, de cadena sencilla o doble, segmentados o continuos, y presentar envoltura lipídica o no hacerlo. Estas diferencias no solo determinan sus estrategias de replicación, adaptación y evolución, sino también, la forma en que interactúan con las células hospedadoras, evaden la respuesta inmunitaria y desarrollan mecanismos de transmisión. El comprender esta diversidad biológica constituye la base para el diseño de herramientas diagnósticas, vacunas, medicamentos antivirales y estrategias de vigilancia capaces de responder con eficacia a los desafíos que plantea la constante evolución del mundo viral.

La extraordinaria diversidad de los virus también se manifiesta en la complejidad de sus estrategias de replicación. Los virus con genoma de ADN, en su mayoría, utilizan la maquinaria celular del núcleo para multiplicarse, mientras que los virus de ARN suelen replicar su material genético en el citoplasma mediante polimerasas de ARN. Existen, además, grupos con mecanismos aún más sofisticados, como los retrovirus, capaces de sintetizar ADN a partir del ARN mediante la acción de la transcriptasa inversa e integrar posteriormente ese material genético al genoma de la célula huésped. Estas notables estrategias de replicación, no solo explican la capacidad de adaptación y de evolución de los virus, sino que han convertido a estos microorganismos en modelos esenciales para comprender procesos fundamentales de la biología molecular, la genética y la interacción entre los microorganismos y sus hospedadores.

Entre la amplia diversidad viral, los virus respiratorios ocupan un lugar prioritario por su extraordinaria capacidad para generar epidemias y pandemias. Su transmisión eficiente a través de gotas respiratorias, aerosoles o contacto estrecho, facilita su rápida diseminación en las poblaciones susceptibles, fenómeno que se ve amplificado por la intensa movilidad humana y la creciente interconectividad global. En un mundo donde millones de personas cruzan diariamente las fronteras internacionales, un nuevo virus respiratorio puede propagarse entre continentes en cuestión de días y transformar un brote local en una emergencia sanitaria de alcance mundial.

La pandemia de COVID-19 confirmó con contundencia una realidad que la vigilancia epidemiológica había advertido durante décadas: los virus respiratorios, particularmente los coronavirus y los virus de la influenza, constituyen una amenaza permanente para la seguridad sanitaria global. No es casual que la mayoría de las pandemias registradas durante el último siglo hayan sido ocasionadas por agentes virales transmitidos por vía respiratoria. Su gran capacidad de mutación, adaptación y dispersión exige mantener sistemas de vigilancia fuertes, dinámicos y sustentados en el conocimiento científico más avanzado.

El responder eficazmente a estas amenazas exige mucho más que infraestructura tecnológica. Requiere capacidades científicas integrales que abarquen el diagnóstico molecular oportuno, la secuenciación genómica de alta resolución, el análisis bioinformático, la caracterización virológica, la vigilancia epidemiológica y la modelación matemática de la transmisión. Detrás de cada una de estas capacidades existe un elemento común e irremplazable: el talento humano altamente calificado. La formación de especialistas capaces de interpretar, integrar y transformar la información científica en acciones de salud pública, constituye uno de los activos más estratégicos para fortalecer la preparación y la respuesta frente a futuras emergencias sanitarias.

En Colombia, el Instituto Nacional de Salud ocupa un lugar central dentro de esta arquitectura de vigilancia. Como laboratorio nacional de referencia y autoridad científica en salud pública, lidera la confirmación diagnóstica, la vigilancia epidemiológica, la caracterización molecular de los virus circulantes y el fortalecimiento técnico de la Red Nacional de Laboratorios. A través de su Grupo de Virología, el Instituto coordina procesos que permiten detectar oportunamente la circulación de agentes virales, identificar variantes emergentes y generar evidencia científica que orienta la toma de decisiones en salud pública.

El Laboratorio de Virología del Instituto constituye, además, un referente nacional e internacional para la vigilancia de enfermedades como la poliomielitis, la influenza, el sarampión, la rubéola, la fiebre amarilla, la rabia, el dengue, el chikunguña, el Zika, las hepatitis virales y otros eventos de importancia en salud pública. Su participación en redes internacionales de vigilancia, sumada a una sólida agenda de investigación aplicada y de producción científica, fortalece la capacidad del país para anticipar riesgos, responder a brotes y contribuir al conocimiento global sobre los virus emergentes y reemergentes. En un medio en el cual las amenazas infecciosas evolucionan de manera constante, el fortalecimiento de instituciones científicas como el Instituto Nacional de Salud y la formación continua de profesionales altamente especializados, constituyen una inversión estratégica para proteger la salud de las generaciones presentes y futuras.

Las capacidades tecnológicas desarrolladas por el Instituto Nacional de Salud reflejan la evolución que ha experimentado la virología durante las últimas décadas. Herramientas como el aislamiento viral, el cultivo celular, las pruebas de neutralización, la secuenciación de Sanger, la secuenciación de nueva generación, la RT-PCR convencional y la RT-PCR en tiempo real, conforman hoy un ecosistema científico que permite, no solo identificar con precisión los agentes virales conocidos, sino también, detectar oportunamente nuevos agentes patógenos con potencial epidémico o pandémico. Estas plataformas representan mucho más que avances tecnológicos: constituyen la base sobre la cual se sustenta la vigilancia virológica moderna y la capacidad de respuesta de los sistemas de salud pública.

Entre estas herramientas, la reacción en cadena de la polimerasa con transcripción inversa en tiempo real (RT-PCR) se ha consolidado como el estándar de referencia para el diagnóstico de numerosos virus de ARN de importancia médica. Su gran sensibilidad y especificidad permiten detectar el material genético viral desde las primeras etapas de la infección, favoreciendo decisiones oportunas en vigilancia epidemiológica, atención clínica y control de brotes. Detrás de cada resultado existe un proceso especializado que integra la adecuada obtención de muestras, la extracción y purificación del ARN viral, la síntesis de ADN complementario mediante la transcriptasa inversa y la amplificación de secuencias específicas mediante sistemas fluorescentes. La precisión de este procedimiento depende, en última instancia, del conocimiento científico y de la experiencia de los profesionales que lo ejecuten e interpreten.

En Colombia, estas capacidades han sido fortalecidas y consolidadas por el Instituto Nacional de Salud, que ha implementado metodologías moleculares estandarizadas y validadas para la vigilancia de virus respiratorios y de otros agentes emergentes de importancia en salud pública. La aplicación de estrictos controles de calidad y la participación permanente en los programas internacionales de evaluación externa garantizan la confiabilidad de los resultados y le permiten al país responder con rapidez y rigor científico frente a brotes, epidemias y potenciales pandemias. No obstante, la eficacia de estos sistemas no depende exclusivamente de la disponibilidad de tecnología, sino de la existencia de equipos humanos capaces de innovar, de interpretar la evidencia y de adaptarse a un panorama epidemiológico en constante transformación.

Es precisamente en este contexto, en el que la formación doctoral adquiere un carácter estratégico. La complejidad creciente de los desafíos sanitarios exige investigadores capaces de trascender la aplicación rutinaria de técnicas diagnósticas para generar conocimiento original, liderar procesos de innovación y transformar los hallazgos científicos en soluciones concretas para la salud pública. El formar doctores en virología significa fortalecer la capacidad del país para comprender la dinámica evolutiva de los virus, desarrollar nuevas metodologías diagnósticas, impulsar la vigilancia genómica y contribuir a la formulación de políticas públicas sustentadas en evidencia científica.

La alianza entre el Instituto Nacional de Salud y la Universidad Simón Bolívar representa, en este sentido, una visión compartida de futuro. Más que un programa académico, constituye una apuesta estratégica por consolidar una masa crítica de investigadores capaces de responder a los desafíos que plantea la virología contemporánea. Áreas como la vigilancia genómica, la biología molecular de los virus, la interacción huésped-patógeno, la epidemiología molecular, el diagnóstico avanzado y la preparación frente a pandemias, requieren profesionales con una formación científica de excelencia, capaces de liderar investigaciones de alto impacto y de integrarse a las principales redes internacionales de generación de conocimiento.

El camino hacia la formación doctoral supone importantes desafíos. Implica acceder a recursos para investigación, formarse en laboratorios de alta complejidad, dominar el lenguaje de la ciencia global, publicar en revistas de gran impacto y participar activamente en comunidades científicas internacionales. En muchos casos, también exige movilidad académica, adaptación a nuevos entornos de investigación y una permanente actualización frente al acelerado avance del conocimiento. Sin embargo, estos retos representan, al mismo tiempo, una oportunidad para consolidar una comunidad científica más sólida, conectada y competitiva.

Los beneficios de esta apuesta trascienden ampliamente el ámbito académico. Cada investigador formado fortalece la capacidad nacional para producir conocimiento, desarrollar tecnologías diagnósticas, innovar en estrategias de vigilancia y contribuir a la formulación de decisiones sustentadas en la evidencia. Asimismo, incrementa la autonomía científica y tecnológica del país, reduce la dependencia de desarrollos externos y favorece la construcción de respuestas adaptadas a las necesidades epidemiológicas de Colombia y de la región.

La historia demuestra que las pandemias no constituyen episodios aislados, sino fenómenos recurrentes que han acompañado la evolución de la humanidad y continuarán representando uno de los principales desafíos para la salud global. La inmensa diversidad viral, la permanente aparición de nuevos agentes patógenos y la capacidad de los virus respiratorios para propagarse rápidamente en las poblaciones susceptibles, obligan a fortalecer de manera sostenida las capacidades científicas y tecnológicas de los países. La experiencia acumulada con la viruela, las pandemias de influenza, el SARS, el MERS y la COVID-19, ha dejado una lección inequívoca: la preparación frente a futuras amenazas no comienza cuando emerge una crisis, sino mucho antes, mediante la inversión continua en ciencia, innovación y formación de talento humano.

En estas circunstancias, la formación doctoral en virología trasciende el ámbito universitario para convertirse en una política estratégica de Estado. El invertir en investigadores altamente calificados significa fortalecer la vigilancia epidemiológica, acelerar la generación de conocimiento, impulsar la innovación tecnológica y consolidar la capacidad del país para anticipar, detectar y responder a los desafíos que plantean los virus emergentes y reemergentes. En un mundo donde la próxima pandemia no es una posibilidad remota, sino una certeza biológica, el conocimiento continúa siendo la herramienta más poderosa para proteger la vida y garantizar la seguridad sanitaria de las generaciones presentes y futuras.

En este contexto, la consolidación de programas de formación doctoral en virología se convierte en una prioridad estratégica para fortalecer la seguridad sanitaria de Colombia y de Latinoamérica. Las lecciones que dejaron las grandes epidemias y pandemias de los dos últimos siglos, evidencian que la capacidad de respuesta de un país no depende únicamente de su infraestructura o de la disponibilidad de tecnologías diagnósticas, sino, principalmente, del conocimiento y del talento humano capaz de anticipar, comprender y enfrentar amenazas biológicas cada vez más complejas.

La alianza entre el Instituto Nacional de Salud, como laboratorio nacional de referencia y máxima autoridad científica en salud pública, y la Universidad Simón Bolívar, reconocida por su compromiso con la formación avanzada y la investigación de excelencia, representa una oportunidad sin precedentes para consolidar una nueva generación de científicos con las competencias necesarias para liderar la investigación, la vigilancia y la innovación en virología. Esta iniciativa responde a una necesidad nacional y regional: fortalecer las capacidades científicas para enfrentar un medio epidemiológico caracterizado por la rápida aparición de agentes patógenos emergentes, la expansión de enfermedades favorecida por el cambio climático, la globalización y la creciente interacción entre los seres humanos, los animales y los ecosistemas.

El invertir en la formación doctoral en virología trasciende el propósito de formar investigadores altamente calificados. Significa fortalecer la soberanía científica del país, reducir la dependencia del conocimiento y de las tecnologías desarrolladas en otras regiones, consolidar capacidades nacionales para generar evidencia y promover respuestas oportunas frente a epidemias y pandemias. En esencia, representa una apuesta por la autonomía científica, la innovación y la construcción de soluciones adaptadas a las realidades sanitarias de Colombia y Latinoamérica.

La *Formación doctoral en virología*, concebida de manera conjunta por el Instituto Nacional de Salud y la Universidad Simón Bolívar, refleja una visión de futuro en la que la ciencia se reconoce como un pilar de desarrollo y de la salud pública. Más que un programa académico, constituye un proyecto de país orientado a consolidar liderazgo científico regional en una disciplina que será determinante para afrontar los desafíos sanitarios del siglo XXI. El formar doctores en virología implica formar investigadores capaces de ampliar las fronteras del conocimiento, desarrollar nuevas herramientas diagnósticas, impulsar la vigilancia genómica, fortalecer la investigación traslacional, y generar evidencia científica que respalde las decisiones con impacto directo sobre la vida y el bienestar de las poblaciones.

Para Colombia, este programa representa un hito en el fortalecimiento de sus capacidades científicas y tecnológicas; para Latinoamérica, constituye una oportunidad para consolidar una masa crítica de expertos capaces de liderar desde la región, la investigación y la respuesta frente a futuras amenazas biológicas. En un contexto en el que la salud global exige respuestas cada vez más integradas, colaborativas y sustentadas en la evidencia, la formación de investigadores de excelencia deja de ser una aspiración académica para convertirse en un componente esencial de la seguridad sanitaria y del desarrollo sostenible.

La convergencia entre el Instituto Nacional de Salud y la Universidad Simón Bolívar envía, además, un mensaje contundente sobre el papel transformador de la ciencia cuando las instituciones unen capacidades, conocimiento y visión de largo plazo. Cada estudiante que emprenda este doctorado asumirá el compromiso de contribuir al avance de la virología, fortalecer la salud pública y ampliar las posibilidades de protección para las generaciones futuras. Su formación será, al mismo tiempo, un aporte al conocimiento universal y una respuesta concreta a las necesidades de nuestros territorios.

Los virus continuarán evolucionando, adaptándose y desafiando los sistemas de salud. Esa es una constante de la historia. Lo que sí puede cambiar es nuestra capacidad para comprenderlos, anticiparnos a su comportamiento y responder con mayor eficacia. Esa capacidad no surge de manera espontánea durante una crisis; se construye mediante una inversión sostenida en ciencia, educación e innovación.

Por ello, la *Formación doctoral en virología* constituye mucho más que un programa de posgrado: representa una inversión estratégica en el futuro, una apuesta por la soberanía científica, un compromiso con la excelencia académica y una contribución decisiva al fortalecimiento de la salud pública. En última instancia, el formar científicos es configurar la primera línea de defensa frente a las amenazas biológicas del mañana y reafirmar que el conocimiento seguirá siendo la herramienta más poderosa para proteger la vida, promover el bienestar y garantizar un futuro más seguro para Colombia, Latinoamérica y el mundo.
